# An evaluation of multi-probe locality sensitive hashing for computing similarities over web-scale query logs

**DOI:** 10.1371/journal.pone.0191175

**Published:** 2018-01-18

**Authors:** Graham Cormode, Anirban Dasgupta, Amit Goyal, Chi Hoon Lee

**Affiliations:** 1 Department of Computer Science, University of Warwick, Coventry, United Kingdom; 2 Computer Science and Engineering, IIT Gandhinagar, Gandhinagar, India; 3 Yahoo Research, Sunnyvale CA, United States of America; Kaohsiung Medical University, TAIWAN

## Abstract

Many modern applications of AI such as web search, mobile browsing, image processing, and natural language processing rely on finding similar items from a large database of complex objects. Due to the very large scale of data involved (e.g., users’ queries from commercial search engines), computing such near or nearest neighbors is a non-trivial task, as the computational cost grows significantly with the number of items. To address this challenge, we adopt Locality Sensitive Hashing (a.k.a, LSH) methods and evaluate four variants in a distributed computing environment (specifically, Hadoop). We identify several optimizations which improve performance, suitable for deployment in very large scale settings. The experimental results demonstrate our variants of LSH achieve the robust performance with better recall compared with “vanilla” LSH, even when using the same amount of space.

## 1 Introduction

Every day, hundreds of millions of people visit web sites and commercial search engines to pose queries on topics of their interest. Such queries are typically just a few key words intended to specify the topic that the user has in mind. To provide users with a high quality service, search engines such as Bing, Google, and Yahoo require intelligent analysis to realize users’ implicit intents. The key resource that they have to help tease out the intent is their large history of requests, in the form of large scale query logs, as well as the log of user actions on the corresponding result pages. A key primitive in learning users’ intents is finding the nearest neighbors for a user-given query. Computing nearest neighbors is useful for many search-related problems on the Web and Mobile such as finding related queries [1–3], finding near-duplicate queries [4], spelling correction [5, 6], and diversifying search results [7]; and Natural Language Processing (NLP) tasks such as paraphrasing [8, 9], calculating distributional similarity [10–12], and creating sentiment lexicons from large-scale Web data [13].

In this paper, we focus on the problem of finding nearest neighbors over very large data sets, and ground our study with the application of searching for the best match of a given query from very large scale query logs from a large search engine. In order to understand the implicit users’ intent, each query is initially represented in a high dimensional feature space, where each dimension corresponds to a clicked url. Given the importance of this question, it is critical to design algorithms that can scale to many queries over huge logs, and allow online and offline computation. However, computing nearest neighbors of a query can be very costly. Naive solutions that involve a linear search of the set of possibilities are simply infeasible in these settings due to the computational cost of processing hundreds of millions of queries. Even though distributed computing environments such as Hadoop make it feasible to store and search large data sets in parallel, the naive pairwise computation is still infeasible. The reason is that the total amount of work performed is still huge, and simply throwing more resources at the problem is not effective. Given a log of hundreds of millions queries, most are “far” from a query of interest, and we should aim to avoid doing many “useless” comparisons that only confirm that other queries are indeed far from it.

In order to address the computational challenge, this paper aims to find nearest neighbors by doing a *small* number of comparisons—that is, sublinear in the dataset size—instead of brute force linear search. In addition to minimizing the number of comparisons, we aim to retrieve neighboring candidates with 100% precision and high recall. It is important that the false positive rate (ratio of “incorrectly” identifying queries as close) is penalized more severely than the false negative rate (ratio of missing “true” neighbors).

When seeking exact matches for queries, effective solutions are based on storing values in a hash table and mapping in via hash functions. The generalization of this approach to approximate matches is the framework of Locality Sensitive Hashing, where queries are more likely to collide under the hash function if they are more alike, and less likely to collide if they are less alike. The methods we propose in this paper meet our criteria by extending Locality Sensitive Hashing [14–16]. In particular, we apply the framework within a distributed system, Hadoop, and take advantage of its distributed computing power.

Our work makes the following contributions:

1. We describe four variants of vanilla LSH motivated by the research on Multi-Probe LSH [17]. We show that two of these achieve much better recall than vanilla LSH using the same number of hash tables. The main idea behind these variants is to intelligently probe multiple “nearby” buckets within a table that have high probability of containing near neighbors of a query.
2. We present a framework on Hadoop that efficiently finds nearest neighbors for a given query from commercial large-scale query logs in sublinear time.
3. We discuss the applicability of our framework on two real-world applications: finding related queries and removing (near) duplicate queries. The algorithms presented in this paper are currently being implemented for production use within a large search provider.

## 2 Problem statement

We start with user query logs *C* having query vectors collected from a commercial search engine over some domain (e.g. URLs); closeness of queries is measured via cosine similarity on the corresponding vectors. Given a set of queries *Q* and similarity threshold *τ*, the problem is to develop a batch process to return a *small* set *T* of candidate neighbors from *C* for each query *q* ∈ *Q* such that:

1. *T* = {*l* ∣ *s*(*l*, *q*) ≥ *τ*, *l* ∈ *C*}, where *s*(*q*_1_, *q*_2_) is a function to compute a similarity score between query feature vector *q*_1_ and *q*_2_;
2. *T* achieves 100% precision with “large” recall. That is, our aim is to achieve high recall, while using a scalable efficient algorithm.

The exact brute force algorithm to solve the above problem would be to compute *s*(*l*, *q*) for all *q* ∈ *Q* and all *l* ∈ *C* and return those (*l*, *q*) where *s*(*l*, *q*) > *τ*. This approach is computationally infeasible on a single machine, even if the size of *Q* is of the order of few thousands when the size of *C* is hundreds of millions. Even in a distributed setting such as Hadoop, the resulting communication needed between machines makes this strategy impractical.

Our aim is to study locality sensitive hashing techniques that enable us to return a set of candidate neighbors while performing a much smaller (*sublinear* in |*Q*| × |*C*|) set of comparisons. In order to tackle this scalability problem, we explore the combination of distributed computation using a map-reduce platform (Hadoop) as well as locality sensitive hashing (LSH) algorithms. We explore a few commonly known variants of LSH and suggest several variants that are suitable to the map-reduce platform. The methods that we propose meet the practical requirements of a real life search engine backend, and demonstrates how to use locality sensitive hashing on a distributed platform.

## 3 Proposed approach

We describe a distributed Locality Sensitive Hashing framework based on map-reduce. First, we present the “vanilla” LSH algorithm due to Andoni and Indyk [16]. This algorithm builds on prior work on LSH and Point Location in Equal Balls (PLEB) [14, 15]. Subsequent prior work on new variants of PLEB [18] for distributional similarity can be seen as implementing a special case of Andoni and Indyk’s LSH algorithm. We next present four variants of vanilla LSH motivated by the technique of Multi-Probe LSH [17]. A significant drawback of vanilla LSH is that it requires a large number of hash tables in order to achieve good recall in finding nearest neighbors, making the algorithm memory intensive. The goal of Multi-probe LSH is to get significantly better recall than the vanilla LSH with the same number of hash tables. The main idea behind Multi-probe LSH is to look up multiple buckets within a table that have a high probability of containing the nearest neighbors of a query. We present the high-level ideas behind the Multi-probe LSH algorithm; for more details, the reader is referred to [17].

### 3.1 Vanilla LSH

The LSH algorithm relies on the existence of a family of locality sensitive hash functions. Let *H* be a family of hash functions mapping $R^{D}$ to some universe S. For any two query terms *p*, *q*, we choose *h* ∈ *H* uniformly at random and analyze the probability that *h*(*p*) = *h*(*q*). Suppose *d* is a distance function (e.g. cosine distance), *R* > 0 is a distance threshold, and *c* > 1 an approximation factor. Let *P*_1_, *P*_2_ ∈ (0, 1) be two probability thresholds. The family *H* of hash functions is called a (*R*, *cR*, *P*_1_, *P*_2_) locality sensitive family if it satisfies the following conditions:

1. If *d*(*p*, *q*) ≤ *R*, then Pr[*h*(*p*) = *h*(*q*)] ≥ *P*_1_,
2. If *d*(*p*, *q*) ≥ *cR*, then Pr[*h*(*p*) = *h*(*q*)] ≤ *P*_2_

An LSH family is generally interesting when *P*_1_ > *P*_2_. However, the difference between *P*_1_ and *P*_2_ can be very small. Given a family *H* of hash functions with parameters (*R*, *cR*, *P*_1_, *P*_2_), the LSH algorithm amplifies the gap between the two probabilities *P*_1_ and *P*_2_ by concatenating *K* hash functions to create *g*(⋅) as: *g*(*q*) = (*h*_1_(*q*), *h*_2_(*q*), …, *h*_*K*_(*q*)). A larger value of *K* leads to a larger gap between probabilities of collision for close neighbors (i.e. distance less than *R*) and those for neighbors that are far (i.e. distance more than *cR*); the corresponding probabilities are $P_{1}^{K}$ and $P_{2}^{K}$ respectively. This amplification ensures high *precision* by reducing the probability of dissimilar queries having the same hash value. To increase the *recall* of the LSH algorithm, Andoni et al. use *L* hash tables, each constructed using a different *g*_*j*_(⋅) function, where each *g*_*j*_(⋅) is defined as *g*_*j*_(*q*) = (*h*_1,*j*_(*q*), *h*_2,*j*_(*q*), …, *h*_*K*,*j*_(*q*))); ∀1 ≤ *j* ≤ *L*.

**Algorithm 1** Locality Sensitive Hashing Algorithm

**Preprocessing:** Input is *N* queries with their respective feature vectors.

- Select *L* functions *g*_*j*_, *j* = 1, 2, …, *L*, setting *g*_*j*_(*q*) = (*h*_1,*j*_(*q*), *h*_2,*j*_(*q*), …, *h*_*K*,*j*_(*q*)), where {*h*_*i*,*j*_, *i* ∈ [1, *K*], *j* ∈ [1, *L*]} are chosen at random from the LSH family.
- Construct *L* hash tables, ∀1 ≤ *j* ≤ *L*. All queries with the same *g*_*j*_ value (∀1 ≤ *j* ≤ *L*) are placed in the same bucket.

**Query:** Set of *M* test queries. Let *q* denote a test query.

- For each *j* = 1, 2, …, *L*
  - Retrieve all the queries from bucket *g*_*j*_(*q*)
  - Compute cosine similarity between query *q* and all retrieved queries. Return all the queries within threshold *τ*.

### 3.2 LSH for cosine similarity

For cosine similarity we adapt the LSH family defined by Charikar [15]. The cosine similarity between two queries $p,q\in R^{D}$ is $\left(\frac{p.q}{∥p∥∥q∥}\right)$. The LSH functions for cosine similarity use a random vector $\alpha \in R^{D}$ to define a hash function as *h*_*α*_(*p*) = sign(*α* ⋅ *p*). A negative sign is interpreted as 0 and positive sign as 1 to generate indices of buckets in the hash tables (i.e. the range of each *g*_*j*_) as *K* bit vectors. To create *α*, we exploit the intuition in [19] and sample each coordinate of *α* from {−1, +1} with equal probability. In practice, these are generated by hash functions that maps that index to {−1, +1} (a.k.a. the “hashing trick” of [20]). This lets us avoid explicitly storing a (huge) *D* × *K* × *L* random projection matrix.

Algorithm 1 gives the algorithm for creating and querying the data structure. In a preprocessing step, the algorithm takes as input *N* queries along with the associated feature vectors. In our application, each query is represented using an extremely sparse and high dimensional feature vector constructed as follows: for query *q*, we take all the webpages (urls) that any user has clicked on when querying for *q*. Using this representation, we generate the *L* different hash values for each query *q*, where each such hash value is again the concatenation of *K* hash functions. These *L* hash values per query are then used to create *L* hash tables. Since the width of the index of each bucket is *K* and each coordinate is one bit, each hash table contains 2^*K*^ buckets. Each query term is placed in its respective buckets in each of the *L* hash tables.

To retrieve near neighbors, we first find all query terms appearing in the buckets associated with each of the *M* test queries. We compute cosine similarity between each of the retrieved terms and the input test queries and return all those queries as neighbors which are within a similarity threshold (*τ*).

The above algorithm fits the Map-Reduce setting quite naturally. We describe a batch setting which performs the LSH on all queries together to perform an all-pairs comparison; other variations are possible depending on the setting. Our implementation performs two map-reduce iterations: in the first phase, the map jobs read in all the queries and their vector representation and outputs key-value pairs that contain the hash-function id (∈[1, *L*]) and the bucket id (∈[0, 2^*K*^ − 1]) as the keys and the query as the value. The reduce jobs then aggregate all queries belonging to a single bucket for a particular hash function, and output candidate pairs. A second map-reduce job then joins these candidate query pairs with their respective feature vectors, computes the exact cosine similarity, and outputs the pairs that have similarity larger than *τ*, ensuring that our precision is 100%. To only consider matches between the *M* test queries and the *N* stored queries, we simply tag each query with its type (test or stored), and only consider candidate pairs that have one of each type. Our experiments show that this map-reduce implementation scales to hundreds of millions of queries.

### 3.3 Reusing hash functions

Directly implementing vanilla LSH requires *L* × *K* hash functions. But generating hash functions is computationally expensive as it takes time to read all features and evaluate hash functions over all those features to generate a single bit. To reduce the number of hash functions evaluations, we use a trick from Andoni and Indyk [16] in which hash functions are reused to generate *L* tables. *K* is assumed to be even and $R\approx \sqrt{L}$. We generate *f*_*j*_(*q*) = (*h*_1,*j*_(*q*), *h*_2,*j*_(*q*), …, *h*_*K*/2,*j*_(*q*))) of length *k*/2. Next, we define *g*(*q*) = (*f*_*a*_, *f*_*b*_), where 1 ≤ *a* < *b* ≤ *R*. Using such pairings, we can thus generate $L=\frac{R(R-1)}{2}$ hash indices. This scheme requires $O(K\sqrt{L}$) hash functions, instead of *O*(*KL*). We use this trick to generate *L* hash tables with bucket indices of width *K* bits.

### 3.4 Multi-Probe LSH

Since generating hash functions can be computationally expensive and the memory required by the algorithm scales linearly with *L*, the number of hash tables, it is desirable to keep *L* small. The large memory footprint of vanilla LSH makes it impractical for many real applications. Here, we first describe four new variants of the vanilla LSH algorithm motivated by the intuition in Multi-probe LSH [17]. Multi-probe LSH obtains significantly higher recall than vanilla LSH while using the same number of hash tables. The main intuition for Multi-probe LSH is that in addition to looking at the hash bucket that a test query *q* falls in, it is also possible to look at the neighboring buckets in order to find its near neighbor candidates. Multi-probe LSH in [17] suggests exploring neighboring buckets in order of their Hamming distance from the bucket in which *q* falls. They show (empirically) that these neighboring buckets contain the near neighbors with very high probability. Though Multi-probe LSH achieves higher recall for the same number of hash tables, it makes more probes as it searches multiple buckets per table. The advantage of searching multiple buckets over generating more tables is that less memory and time is required for table creation.

The original Multi-probe LSH algorithm was developed for Euclidean distance. However, that algorithm does not immediately translate to our setting of cosine similarity. For example, in generating the list of other buckets inspected, [17] utilizes the distance of the hash value to the bucket boundary—this makes sense when the hash value is a real number, but we have bits. We present four variants of Multi-probe LSH for cosine similarity:

- **Random Flip Q:** Our baseline version first computes the initial LSH of a test query *q* to give the *L* bucket ids. Next, we create *F* alternate bucket ids by flipping a set of coordinates randomly in each *g*_*j*_(*q*). For scalability, we restrict our implementation to flipping a single bit out of the *K* possible bits each time, and ensure that the sampling is done without repetition. Since the hash functions are randomly chosen, we implement this by simply flipping the first bit, then revert it and flipping the second bit, until we reach the *F*’th bit.
- **Random Flip B:** The second variant is another baseline similar to the previous one. Instead of just flipping the bits for only the test query, here we flips bits for *both* the test query *and* all the queries in the database: this increases the “radius” of the search. We treat each database point as if it were a query, and flip a random bit in each of its hash representations *F* times over. Note that this method requires applying flipping to all the queries in the database. This is a one-time operation done while creating the database. We generate up to *F* variants of each hash, so for each query, first its *L* LSH representations of length *K* are generated. On each of the *L* representations, flipping of bits is applied *F* times to generate *LF* representations of a query.
- **Distance Flip Q:** The third variant is a smarter version of Random Flip Q. It selects coordinates based on the *distance* of *q* from the random hyperplane (hash function) used to create this coordinate. The distance of the test query *q* from the random hyperplane *α* is the absolute value which we get before applying the sign function on it (see Section 3.2), i.e., abs(*α* ⋅ *q*), the distance of *q* from hyperplane *α*. This method flips up to *F* coordinates in order of increasing distance from the hyperplane. That is, for each group of *K* hash values, we sort by the distance to the hyperplane, and swap each of the first *F* of these in turn. As with Random Flip Q, we restrict to flip only a single bit in each repetition, so *F* ≤ *K*.
- **Distance Flip B:** Our fourth variant flips bits for both the test query and for the queries in the database (i.e., the intelligent version of the second baseline). Like Random Flip B, it rquires us to flip all database items, which is a one-time data pre-processing step.

The map-reduce implementation of Multi-probe LSH follows the same structure as the vanilla one—the map phase of the first map-reduce job generates the alternate bucket-ids for both the test query and the queries in the database. For all LSH methods, the first preprocessing step is the same, which is to evaluate the hash functions to generate $K\sqrt{L}$ bits. The second step is to generate tables indexed by the hash function id and bucket id. Within the map job, each query is mapped to its various indices. For multiprobe LSH, each query is also mapped to additional indices. Within the reduce job, all queries with the same index are collected and all colliding pair of queries (that share the same index) are output. The final step is to compute similarity for the colliding pairs and only keep those pairs that are above the threshold *τ* (based on exact comparison using their original feature representation).

### 3.5 Time cost

The exact running time of these algorithms is hard to predict, as it depends on the distribution of the data, as well as the configuration of the computing environment (number of machines, communication topology etc.). Broadly speaking, the time cost is comprised of the preprocessing (the one-time cost to build the database of queries), and the runtime cost to process a new set of query look-ups. The communication cost of our algorithms in the Map-Reduce framework is low, since the majority of the work is embarassingly parallel. Across all our methods, at most $O(K\sqrt{L})$ hash function evaluations are needed. While it may seem that the multiprobe LSH methods require more hash function evaluations, we aim to choose the parameters *K* and *L* so that less work is needed overall in order to achieve the same level of recall compared to the vanilla LSH methods. The final step, to compute the true similarity of the retrieved pairs, is proportional to the number of collisions. We expect the proposed methods to be faster here, since there should be fewer candidates to test. This stands in contrast to the naive exact method, which performs an all-pairs comparison.

Due to the variation in real world configurations, we do not explicitly measure the time taken to perform the experiments. Rather, we make use of the number of comparisons as a surrogate. Our informal tests indicate that this is a robust measure of effort required, since the total CPU time was broadly proportional to this measure across a number of different configurations, while we find that the number of comparisons is not subject to interference from external factors (overall cluster loading etc.).

## 4 Experiments

### 4.1 Data

We use two data sources for our experiments. The first is the AOL-logs dataset that contains search queries posed to the AOL search engine and that dataset was made available in 2006 [21]. This data is accessible from the figshare repository, https://doi.org/10.6084/m9.figshare.5527231.v1. We also use a partial sample of query logs from a commercial search engine, denoted as Qlogs. Note that realistic query log information is considered confidential and contains potentially sensitive information about individuals. We are therefore careful in our handling of the data, and report only aggregate results and carefully chosen examples. We do not have permission to share the Qlogs data further, but to allow reproduction of results we show all our analyses on the public data. We were provided access to this data on request to Yahoo via an electronic file. Requests for access to this data can be addressed to Yahoo’s academic relations manager, mailto:kimcapps@oath.com.

As Qlogs reaches hundreds of millions of queries (approximately 600*M* unique queries), we generated multiple datasets from Qlogs by sampling at various rates: Qlogs001 represents a 1% sample, Qlogs010 represents a 10% sample and Qlogs100 represents the entire Qlogs. The smaller datasets are primarily used to explore parameter ranges and identify suitable values that we then use to experiment with the larger dataset. For each query *q*, a feature vector in a high dimensional feature space, denoted as **q** = (*f*_1_, *f*_2_, ⋯, *f*_*D*_), was created by setting *f*_*i*_ to be the click through rate of url *i* when shown in the search results page of search-query *q*. Note that in our real implementation, **q** is represented as a sparse feature vector with only non-zero click-through rate features being present. In a pre-processing step, we remove all queries with at most five clicked urls. Table 1 summarizes the statistics of our query-log datasets.

**Table 1:** Query-logs statistics.

Data	*N*	*D*
AOL-logs	0.3 × 10^6^	0.7 × 10^6^
Qlogs001	6 × 10^6^	66 × 10^6^
Qlogs010	62 × 10^6^	464 × 10^6^
Qlogs100	617 × 10^6^	2.4 × 10^9^

**Test Data.** In all experiments we use a randomly sampled set of 2000 queries *Q*, as the test set. That is, we want to find set *T*, where *T* = {*l*∣*s*(*q*, *q*′) ≥ *τ*}, *s*(*q*, *q*′) is cosine similarity, and *q*′ ∈ *C* for *C* ∈ {Qlogs001, Qlogs010, Qlogs100,AOL-logs}. For most experiments, we set the similarity threshold *τ* = 0.7, meaning that for *q*, candidates *q*′ having cosine similarity of larger than or equal to 0.7 are retrieved.

**Evaluation Metrics.** We use two metrics for evaluation: recall and number of comparisons. The recall of an LSH algorithm measures how well the algorithm can retrieve the *true* similar candidates. The number of comparisons performed by an algorithm is computed as the average number of pairwise comparisons done per test query, and measures the total computation done. The aim is to maximize recall and to minimize the number of comparisons.

### 4.2 Evaluating vanilla LSH

First, we vary the similarity threshold parameter *τ* in the range {0.7, 0.8, 0.9} while fixing *K* = 16 and *L* = 10 for the AOL-logs and Qlogs001 datasets. Table 2 shows that *τ* = 0.9 achieves higher recall than *τ* = 0.7. This is expected as finding near duplicates is actually easier than finding near neighbors that satisfy only a looser similarity criterion. For the rest of this paper, *τ* is set as 0.7 since it represents the more challenging case.

**Table 2:** Varying *τ* with fixed *K* = 16 and *L* = 10.

*τ*	AOL-logs		Qlogs001	
	Comparisons	Recall	Comparisons	Recall
0.7	57	.63	1052	.67
0.8		.84		.81
0.9		.98		.96

In the second experiment, we vary *R* to be in {1, 4, 7, 10}, corresponding to values of *L* of {1, 10, 28, 55}, while fixing *K* = 16 on the AOL-logs and Qlogs001 datasets. Recall that *L* denotes the number of hash tables and *K* is the width of the index of the buckets in the table. Increasing *K* results in increasing precision of the candidate pairs by reducing false positives, but *L* needs to be correspondingly increased in order to maintain good recall (i.e. reduce false negatives). Table 3 shows that increasing *L* leads to better recall, at the cost of more comparisons on both datasets. In addition, large *L* means generating many random projection bits and hash tables which is both time and memory intensive. Hence, we fix *L* = 10, to achieve reasonable recall with a tolerable number of comparisons.

**Table 3:** Varying *L* with fixed *K* = 16 and *τ* = 0.7.

L	AOL-logs		Qlogs001	
	Comparisons	Recall	Comparisons	Recall
1	7	.28	106	.36
10	57	.63	1052	.67
28	152	.77	2908	.78
55	297	.89	5648	.84

Next, we vary *K* in {4, 8, 16} while fixing *L* = 10. As expected, Table 4 shows that increasing *K* reduces the number of comparisons and worsens recall on both datasets. This is intuitive as the larger value of *K* leads to larger gap between probabilities of collision for queries that are close and those that are far. Henceforth, we fix *K* = 16 to have an acceptable number of comparisons.

**Table 4:** Varying *K* with fixed *L* = 10 with *τ* = 0.7.

K	AOL-logs		Qlogs001	
	Comparisons	Recall	Comparisons	Recall
4	112,347	.98	2,29,2670	.96
8	11,008	.90	221,132	.88
16	57	.63	1,052	.67

In the fourth experiment, we fix *L* = 10 and *K* = 16 as determined above, and we increase the size of training data. Table 5 demonstrates that as we increase data size, the number of comparisons done by the algorithm also increase. This result indicates that *K* needs to be tuned with respect to a specific dataset, as a larger *K* will reduce the probability of dissimilar queries falling within the same bucket. *K* and *L* can be tuned by randomly sampling a small set of queries. In this paper, we randomly select 2000 queries to tune parameter *K*.

**Table 5:** Fixed *K* = 16 and *L* = 10 with *τ* = 0.7.

Data	Comparisons	Recall
AOL-logs	57	.63
Qlogs001	1,052	.67
Qlogs010	10,515	.64
Qlogs100	105,126	-

Table 6 shows the best choices of *K* for our datasets. We note that on Qlogs100 the precision/recall cannot be computed, as it was computationally infeasible to find the exact similar neighbors. On our biggest dataset of 600M queries, we set *K* = 24 and *L* = 10. These settings require only 464 comparisons (on average) to find approximate neighbors compared to exact cosine similarity that involves brute force search over all 600M queries.

**Table 6:** Best parameter settings of *K* (minimizing comparisons and maximizing recall) with *L* = 10.

Data	Comparisons	Recall
AOL-logs (*K* = 16)	57	.63
Qlogs001 (*K* = 16)	1,052	.67
Qlogs010 (*K* = 20)	695	.53
Qlogs100 (*K* = 24)	464	-

### 4.3 Evaluating multi-probe LSH

First, we compare flipping *F* bits in the query only. We evaluate two approaches: Random Flip Q and Distance Flip Q. We make several observations from Table 7: 1) As expected, increasing the number of flips improves recall at the expense of more comparisons for both Distance Flip Q and Random Flip Q. 2) The last row of Table 7 shows that when we flip all *K* bits (*F* = 16), Distance Flip Q and Random Flip Q converge to the same algorithm, as expected. 3) We see that Distance Flip Q has significantly better recall than Random Flip Q with a similar number of comparisons. In the second row of the table with *F* = 2, the recall of Distance Flip Q is nine points better than that of Random Flip Q.

**Table 7:** Flipping the bits in the query only with *K* = 16 and *L* = 10 on AOL-logs with *τ* = 0.7.

Method	Random Flip Q		Distance Flip Q	
*F*	Comparisons	Recall	Comparisons	Recall
1	108	.65	106	.72
2	159	.66	*155*	**.75**
5	311	.70	303	.79
10	557	.75	552	.81
16	839	.82	839	.82

Table 8 shows the result of flipping *F* bits in both query and the database. In the second row of Table 8 with *F* = 2, Distance Flip B has thirteen points better recall than Random Flip B with a similar number of comparisons. Comparing across the second row of Tables 7 and 8 shows that flipping bits in both query and database has better recall at the expense of more comparisons. This is expected as flipping both means that we increase our “radius of search” to include queries at distance two (one flip in query, one flip in database), and hence have more queries in each table when we probe. We also compared distance-based flipping with random flipping on different input sizes, and found that distance-based flipping always has much better recall compared to random flipping (for brevity, we omit these numbers).

**Table 8:** Flipping the bits in both the query and the database with *K* = 16 and *L* = 10 on AOL-logs with *τ* = 0.7.

Method	Random Flip B		Distance Flip B	
*F*	Comparisons	Recall	Comparisons	Recall
1	204	.71	192	.80
2	433	.73	*405*	**.86**
5	1557	.86	1475	.93
10	4138	.94	4059	.96
16	5922	.96	5922	.96

We select *F* = 2 as the best parameter setting with goal of maximizing recall by restricting comparisons to a minimum. For better recall at the expense of more comparisons, *F* = 5 can also be selected. However, results in Tables 7 and 8 indicate that *F* > 5 does *not* increase recall significantly while leading to more comparisons.

Table 9 gives the results of both variants of distance-based Multi Probe, i.e. Distance Flip Q and Distance Flip B, on different sized datasets. We present results with the parameters *L* = 10, *F* = 2, and value of *K* chosen as per the values used in the final vanilla LSH experiment. As observed there, flipping bits in both query and the database is significantly better in terms of recall with more comparisons. The second and third row of the table respectively shows that flipping bits in both query and the database has eight points better recall on both Qlogs001 and Qlogs010 datasets. With the goal of maximizing recall with some extra comparisons, we select Distance Flip B as our preferred algorithm. Distance Flip B maximizes recall with few tables and comparisons. On our entire corpus (Qlogs100) with hundreds of millions of queries, Distance Flip B only requires 3,427 comparisons per test query, compared to hundreds of millions of comparisons by the exact brute force algorithm. Distance Flip B returns 9 neighbors on average per given query, averaged over 2000 random test queries. Here, many queries are long, and have few neighbors.

**Table 9:** Best parameter settings of *K* (minimizing comparisons and maximizing recall) with *L* = 10, *F* = 2, *τ* = 0.7.

Method	Distance Flip Q		Distance Flip B	
Data	Comps.	Recall	Comps.	Recall
AOL-logs (*K* = 16)	155	.75	405	.86
Qlogs001 (*K* = 16)	2980	.76	7904	.84
Qlogs010 (*K* = 20)	1954	.64	5242	.72
Qlogs100 (*K* = 24)	1280	-	3427	-

### 4.4 Discussion

Tables 10 and 11 shows some qualitative results for a set of arbitrarily chosen queries. These results are found by applying our system (Distance Flip B with parameters *L* = 10, *K* = 24, and *F* = 2) on Qlogs100. These results help to highlight several applications that can take significant advantage of the approximate Distance Flip B algorithm presented in this paper. For example, the second column in Table 10 shows that the returned approximate similar neighbors can be useful in finding related queries [1, 2]. The first column in Table 11 shows an example where we find several popular spelling errors automatically, which can usefully be used for query suggestion.

**Table 10:** 10 similar neighbors returned by Distance Flip B with *L* = 10, *K* = 24, and *F* = 2 on Qlogs100 for two example queries.

how lbs in a ton	coldwell banker baileys harbor
how much lbs is a ton	coldwell banker sturgeon bay wi
number of pounds in a ton	coldwell banker door county
how many lb are in a ton	door county wi mls listings
How many pounds are in a ton?	door county realtors sturgeon bay
how many pounds in a ton	DOOR CTY REAL
1 short ton equals how many pounds	door county coldwell banker
how many lbs in a ton?	door realty
how many pounds in a ton?	coldwell banker door county horizons
How many pounds are in a ton	door county coldwell banker real estate
how many lb in a ton	coldwell banker door county wisconsin

**Table 11:** 10 similar neighbors returned by Distance Flip B with *L* = 10, *K* = 24, and *F* = 2 on Qlogs100 for two example queries.

michaels	trumbull ct weather
maichaels	trumbull ct weather forecast
machaels	weather in trumbull ct
mechaels	weather in trumbull ct 06611
miachaels	trumbull weather forecast
michaeils	trumbull ct 06611
michaelos	trumbull weather ct
michaeks	trumbull ct weather report
michaeels	trumbull connecticut weather
michaelas	weather 06611
michae;ls	weather trumbull ct

One interesting application of near-neighbor finding is to understand specific intents behind the user query. Given a user’s query, Bing, Google, and Yahoo often delivers direct display results that summarize expected contents of the query. For instance, when a query “f stock price” is issued to search engines, the quick summary of the stock quote with a chart is delivered to the user as the part of the search engine result page. Such direct display results are expected to reduce the number of unnecessary clicks by providing the user with the appropriate content early on. However, when the query “f today closing price” is issued to search engines, the three major search engines fail to deliver the same direct display experience to the user query, even though its query intent is strongly related to “f stock price”. By employing an algorithm similar to Distance Flip B, we can build a synonym database, which will help trigger the same direct display among related queries. The first column of Table 10 and the second column of Table 11 show examples of near-duplicate queries that can be automatically answered [4].

Another application is to remove duplicated instances in a set of suggested results. When a query set is retrieved from a repository and presented to users, it is important to remove similar queries from the set so that the user is not distracted by duplicated results. Given a set of queries, we can apply Distance Flip B algorithm to build a lookup table of near-duplicates in order to find the “duplicated query terms” efficiently. As “near-duplicates” among query terms typically require a “higher” degree of similarity (relatively easier problem) than “relatedness”, we can tune parameters (*K*, *L*, *F*) based on a specific *τ* (e.g *τ* = 0.9) from training samples. The second column in Table 11 illustrates several effective duplicates: “trumbull weather ct” and “weather in trumbull ct”.

## 5 Related work

There has been much work in last decade focusing on approximate algorithms for finding similar objects, too much to survey in full, so we highlight some important related publications. From the NLP community, prior work on LSH for noun clustering [10] applied the original version of LSH based on Point Location in Equal Balls (PLEB) [14, 15]. The disadvantage of vanilla LSH algorithm is that it involves generating a large number of hash functions (in the range *L* = 1000) and sorting bit vectors of large width (*K* = 3000). To address that issue, Goyal et al. [18] proposed a new variant of PLEB that is faster than the original LSH algorithm but that still requires large number of hash functions (*L* = 1000). In addition, their work can be seen as an implementing a special case of Andoni and Indyk’s LSH algorithm, that was applied to the problem of detecting new events from a stream of Twitter posts [22].

A major distinction of our research is that existing work deals with approximating cosine similarity by Hamming distance [10, 18, 23–25]. Moran et al. [25] proposed a data-driven non-uniform bit allocation across hyperplanes that uses fewer bits than many existing LSH schemes to approximate cosine similarity by Hamming distance. In all these existing problem settings, the goal is to minimize both false positives and negatives. However, we focus on minimizing false negatives with zero tolerance for false positives. [26] developed a distributed version of the LSH algorithm, for the Jaccard distance metric, that scales to very large text corpora by virtue of being implemented on a map-reduce, and by using clever sampling schemes in order to reduce the communication cost. Our work addresses the cosine similarity metric, and uses bit flipping in a distributed manner to reduce the number of hash tables in LSH and hence the memory.

Other work in this area has addressed engineering throughput for massively parallel computation [27], distributed LSH for Euclidean distance [28], and variants such as “entropy-based LSH”, also for Euclidean distance [29].

## 6 Conclusion

In this work, we applied the vanilla LSH algorithm of Andoni et al. to search query similarity applications. We proposed four variants of LSH that aim to reduce the number of hash tables used. Two of our variants achieve significantly better recall than vanilla LSH while using the same number of hash tables. We also present a framework on Hadoop that efficiently finds nearest neighbors for a given query from a commercial large-scale query logs in sublinear time. On our entire corpus (Qlogs100) with hundreds of millions of queries, Distance Flip B only requires 3,427 comparisons compared to hundreds of millions of comparisons by exact brute force algorithm. In future, we plan to extend our LSH framework to several large-scale NLP, search, and social media applications.
